# Differential inhibition of PDKs by phenylbutyrate and enhancement of pyruvate dehydrogenase complex activity by combination with dichloroacetate

**DOI:** 10.1007/s10545-014-9808-2

**Published:** 2015-01-20

**Authors:** Rosa Ferriero, Clara Iannuzzi, Giuseppe Manco, Nicola Brunetti-Pierri

**Affiliations:** 1 Telethon Institute of Genetics and Medicine, Via Campi Felgrei, 34, 80078 Pozzuoli, Naples Italy; 2 Institute of Protein Biochemistry (IBP), Naples, Italy; 3 Department of Biochemistry, Biophysics and General Pathology, Second University of Naples, Naples, Italy; 4 Department of Translational Medicine, Federico II University of Naples, Naples, Italy

## Abstract

Pyruvate dehydrogenase complex (PDHC) is a key enzyme in metabolism linking glycolysis to tricarboxylic acid cycle and its activity is tightly regulated by phosphorylation catalyzed by four pyruvate dehydrogenase kinase (PDK) isoforms. PDKs are pharmacological targets for several human diseases including cancer, diabetes, obesity, heart failure, and inherited PDHC deficiency. We investigated the inhibitory activity of phenylbutyrate toward PDKs and found that PDK isoforms 1-to-3 are inhibited whereas PDK4 is unaffected. Moreover, docking studies revealed putative binding sites of phenylbutyrate on PDK2 and 3 that are located on different sites compared to dichloroacetate (DCA), a previously known PDK inhibitor. Based on these findings, we showed both in cells and in mice that phenylbutyrate combined to DCA results in greater increase of PDHC activity compared to each drug alone. These results suggest that therapeutic efficacy can be enhanced by combination of drugs increasing PDHC enzyme activity.

**Electronic supplementary material:**

The online version of this article (doi:10.1007/s10545-014-9808-2) contains supplementary material, which is available to authorized users.

## Introduction

Pyruvate dehydrogenase complex (PDHC; E.C. 1.2.4.1) is a key enzyme in metabolism that catalyzes oxidative decarboxylation of pyruvate to produce acetyl-CoA thus linking glycolysis to tricarboxylic acid cycle. PDHC is a large enzyme complex organized around a structural core formed by dihydrolipoamide acetyltranferase (E2 protein) and E3-binding protein (E3BP). Multiple copies of α2β2 heterotetramer pyruvate decarboxylase (E1), the dihydrolipoamide dehydrogenase (E3), and one to two copies each of pyruvate dehydrogenase kinases (PDK; E.C. 2.7.11.2) and pyruvate dehydrogenase phosphatases (PDP; E.C. 3.1.3.43) are non-covalently bound to the E2/E3BP core. PDKs bind via interactions to the inner lipoyl domain (L2) of the E2 subunit (Liu et al 1995; Hiromasa et al 2004; Roche and Hiromasa 2007). PDHC activity is tightly regulated by reversible phosphorylation and dephosphorylation. Phosphorylation of E1α at three specific serine sites (Ser203-α, Ser264-α, Ser271-α) by four mitochondrial PDK isoforms (PDK1, PDK2, PDK3, and PDK4) inactivates the complex whereas dephosphorylation by PDP1 and PDP2 restores enzyme activity. Phosphorylation of only one site renders the enzyme inactive but the physiological significance of the three phosphorylation sites, as well as the need of multiple PDK isoenzymes, has yet to be elucidated. PDHC activity is regulated by transcriptional levels of PDKs and PDPs under different nutritional or disease states. PDK4 and to a lesser extent PDK2 are upregulated in tissues under starvation and diabetes whereas PDPs are downregulated. As a result, under these conditions PDHC activity is reduced for glucose conservation (Harris et al 2001).

PDKs are pharmacological targets for several human diseases including cancer, diabetes, obesity, heart failure, and inherited PDHC deficiency. PDK isoforms are expressed in most tissues and based on similarity of their catalytic domains (Bowker-Kinley and Popov 1999), are grouped within the ATPase/kinase superfamily (composed of bacterial histidine protein kinase, DNA gyrases, and molecular chaperone HSP90). Members of this superfamily share four conserved motifs forming a unique ATP-binding fold (Dutta and Inouye 2000) that includes the ATP lid, whose conformational change is coupled to both ATP hydrolysis and protein-protein interactions (Machius et al 2001). The four PDK isoforms belong to the family of mitochondrial protein kinases that includes the branched-chain α-ketoacid dehydrogenase kinase (BDK; EC 2.7.11.4) in which motifs that normally occur in eukaryotic Ser/Thr/Tyr kinases are absent (Manning et al 2002). Structural studies of PDKs and BDK have revealed that these kinases consist of two distinct domains (Machius et al 2001): the N-terminal regulatory domain formed by eight α-helices with a four-helix bundle-like structure forming the core and the C-terminal catalytic domain containing the phosphotransfer catalytic site that is conserved in the ATPase/kinase superfamily (Dutta and Inouye 2000).

The primary structures of the four PDKs are conserved with 66-74 % identity (Popov et al 1994). PDKs differ in their catalytic activity, responsiveness to modulators such as NADH and acetyl-CoA, and tissue-specific expression (Bowker-Kinley and Popov 1999). PDK1 is highly expressed in heart, PDK2 is ubiquitously expressed, PDK3 has a relatively limited tissue distribution (mostly in testis and to a lesser extent in brain, lung, and kidney), and PDK4 is expressed in heart and skeletal muscle (Gudi et al 1995; Bowker-Kinley et al 1998). PDK2 is expressed at higher levels compared to other isoenzymes, suggesting that it may be the major isoform responsible for regulation of PDHC enzyme activity (Gudi et al 1995). Each PDK isoform exhibits different specificity for the three E1α serine sites; sites 1 and 2 are phosphorylated by all four isoforms whereas site 3 is only modified by PDK1 (Kolobova et al 2001; Korotchkina and Patel 2001a, b). PDK3 binds to L2 of the E2 protein most tightly among the four PDK isoforms.

We have previously shown that phenylbutyrate inhibits PDHC inactivation by competing for binding of E1α to PDK2 through a competitive inhibition with an experimentally measured K_i_ of 0.33 ± 0.08 mM (Ferriero et al 2013). Through a docking simulation, we also identified two putative binding sites of phenylbutyrate on PDK2: one site was found near the ATP lid and the other at the base of the four helix cluster corresponding to the binding site of Pfz3, an allosteric inhibitor of PDK2 (Knoechel et al 2006; Ferriero et al 2013). The goal of this study was to investigate phenylbutyrate inhibiting activity toward the other PDK isoenzymes (PDK1, 3, and 4) and the relative drug binding sites. The identification of phenylbutyrate binding sites on PDKs led us to hypothesize that a combined therapy might be more effective than mono-therapy at increasing enzyme activity for therapeutic applications.

## Materials and methods

### Pyruvate dehydrogenase kinase activity assay

Recombinant human PDK1, PDK3, and PDK4 (Sigma-Aldrich), and E1α [(30–390) plus His tag; molecular weight: 47 KDa] (Sigma-Aldrich) were used for the assays. PDK activity was measured in duplicate as the initial rate of incorporation of [^32^P]-phosphate into E1α with 0.2 mM [*γ*-^32^P]ATP (150–500 cpm/pmol) at 30 °C (Rahmatullah and Roche 1985; Ravindran et al 1996; Baker et al 2000). The activity measured at different time points (30-60-90-120 sec) and protein concentrations (from 50 ng to 800 ng) was established as linear relative to time and protein concentrations. The assay used 0.02 μg of PDKs and measured incorporation after 45 seconds of reaction time. The assay was conducted in a total volume of 50 μl with a buffer A with a final pH of 7.4 and the following composition: 113 mM HEPES-Tris pH 7.4, 60 mM KCl, 30 mM K-HEPES, 2 mM MgCl_2_, 0.2 mM EDTA. The assay mixture also contained 2 mM dithiothreitol. Concentrated protein components (8-6-3-2-1-0.5 μg of E1α) were pre-incubated for 60 minutes at 4 °C in the buffer in which they were prepared and then were added to reaction mixtures for 2 minutes at 30 °C prior to initiation of PDK activity. Phenylbutyrate was added at the concentration of 0.25 mM, 0.5 mM, or 1 mM and then the proteins were incubated in buffer A at 22 °C for 60 seconds, and 0.2 mM of [γ-^32^P]ATP was added. The reaction was terminated by adding 2 mM of ATP, and labeled E1α was separated from unbound ATP by loading 35 μl of mixture onto G-25 Sephadex gel filtration columns (10 × 0.45 mm). The reaction mixtures eluted at the void volume (around 200 μl) were applied to dry paper (Whatman 3MM, 22 mm) previously soaked in 10 % (w/v) trichloroacetic acid. The assay was completed by reading incorporated cpm in vials containing liquid scintillation cocktail in a Beckman LS6500 multi-purpose scintillation counter. Data were analyzed with GraFit Data Analysis Software version 5.0.

### Docking of phenylbutyrate on PDK2 and PDK3

Protein-ligand docking simulations were performed using AutoDock Vina tool (Trott and Olson 2010). The initial PDK models were generated by building hydrogen atoms for the crystal structure of human PDK3 radicicol-bound (PDB chain ID 2Q8I) and human PDK2 ADP-bound (PDB chain ID 2BU8) and by adding Gasteiger charges. An initial conformation of the ligands [phenylbutyrate and dichloroacetate (DCA)] was generated by Cartesian optimization of the ligand model in GROMOS87 force field (PRODRG at http://davapc1.bioch.dundee.ac.uk/prodrg/submit2.html). All side chains and the backbone of the protein were kept rigid as in the crystal structure. Docking was performed first by placing the ligand in a random position by centering the grid on the macromolecule and setting the grid with a 1-Å spacing on the entire protein; after the identification of the best binding sites, further analysis was performed by starting with the ligand in the binding pockets and setting the grid with a 0.375-Å spacing. The affinity expressed in kcal/mol was calculated as the difference in free energy of binding (ΔG) between the protein and the complex. Control of docking procedure was obtained by docking DCA on the PDK2 structure (PDB chain ID 2BU8) after ligand and potassium ion removal and by docking phenylbutyrate on the Val62Leu mutant PDK2 model obtained using the tools and procedures available under the deepView/Swiss-Pdb Viewer program (v 4.1.0). Results were visualized using PyMol (The PyMOL Molecular Graphics System, Version 1.5.0.4 Schrodinger, LLC) wherein conformations for each ligand were found to be within the cavity of protein indicating that the docking run was free from errors.

### Mouse studies

Mouse studies were approved by the Italian Ministry of Health. Phenylbutyrate (Ammonaps, Swedish Orphan Internaltional Lab), phenylacetate (Sigma), DCA (Sigma), or saline were given orally to C57BL/6 mice (Charles River Laboratories) by gavage into doses divided in three daily administrations for three consecutive days (at least n = 5 mice per group). After three days of treatment, animals were sacrificed and brain, liver, and muscle were harvested after cardiac perfusion with phosphate buffered saline (PBS). Crude mitochondria were purified from tissues as previously reported and assayed for PDHC activity as described below (Ferriero et al 2013).

### PDHC enzyme assay and western blots

Human control fibroblasts from normal subjects (BA1020 and NA489) and fibroblasts from a male patient with PDHC deficiency carrying the p.N135S mutation in the *PDHA1* gene (Ferriero et al 2013) were cultured in Dulbecco’s modified Eagle’s medium and 1 % fetal bovine serum.

For PDHC enzyme assays, cultured skin fibroblasts were harvested with trypsin solution and centrifuged. After centrifugation, the pellets were washed twice with PBS. The cell pellet was resuspended in 2 ml buffer A (MOPS, 20 mM KOH pH 7.4, 250 mM sucrose) and 0.2 mg of digitonin for every ml of buffer A. The solution was mixed and kept on ice for 5 minutes and centrifuged at 5000 × g for 3 minutes. The supernatant was mixed with 3 ml buffer B (MOPS, KOH 20 mM pH 7.4, sucrose 250 mM, EDTA Na_4_ 1 mM), kept on ice for 5 minutes, then centrifuged at 10,000 × g for 3 minutes. The supernatants were discarded, and pellets were resuspended in 0.5 ml of 20 mM K-phosphate buffer pH 7.4, and then frozen in liquid nitrogen and thawed at 37 °C three times. PDHC enzyme activity was measured in mitochondrial fractions as described previously (DeVivo et al 1979). Briefly, the assay mixture contained 30 mM HEPES-KOH, 10 mM β-mercaptoethanol, 1 mM CoASH, 0.1 mM NAD, 0.3 mM thiamine pyrophosphate, 0.02 % Triton, 10 mM MgCl_2_, and 1 mM CaCl_2_. The enzyme reaction was initiated with 20 mM of ^14^C-pyruvate sodium (Perkin Elmer) and incubated for 10 minutes at 37 °C. Reaction was terminated with 250 μl of 1 N HCl on ice and left at 37 °C for 60 minutes. The ^14^CO_2_ released from the reaction was captured onto filter paper and transferred in scintillation liquid overnight; the cpm were counted by a β-counter (Beckman LS6500). PDHC enzyme activity was expressed as nmol/min/mg protein. Mitochondria fractions quantified for protein concentrations by the Bradford method (Bradford 1976) were resolved by SDS-PAGE and transferred onto a PVDF membrane. Western blotting analyses with 10 μg of mitochondrial fraction were performed with PhosphoDetect anti-PDH-E1α (p-Ser264-α; Calbiochem AP1062), anti-PDH-E1α (Abcam ab110416), and HRP-conjugated secondary antibodies (GE Healthcare) diluted in 5 % milk in tris-buffered saline plus Tween 20 (TBST) and in 1 % BSA in TBST. Bands were visualized with a chemiluminescence detection system (Pierce) and quantified with Quantity One 1-D Analysis Software version 4.6.7 (Bio-Rad Laboratories).

### Statistical analyses

Statistical significance was computed using the Student’s 2 tail test. A p-value < 0.05 was considered statistically significant.

## Results

**Fig. 1:**
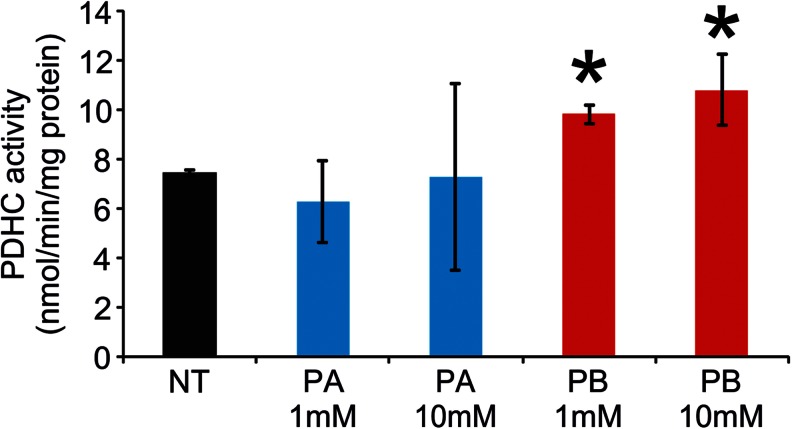
PDHC activity in cells treated with phenylacetate or phenylbutyrate. PDHC activity in wild-type human fibroblasts incubated with 1 mM or 10 mM of phenylbutyrate (red) or phenylacetate (blue) for 24 hours, or untreated (black). Averages ± SD are shown; * p < 0.05. *Abbreviations*: *NT*= not treated (no drug); *PA*= phenylacetate; *PB*= phenylbutyrate

**Fig. 2:**
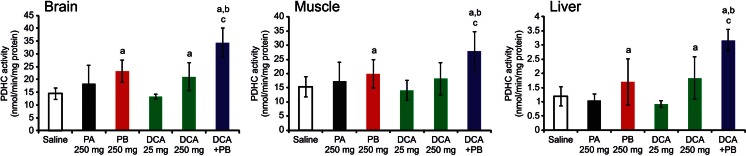
PDHC activity in tissues of mice treated with phenylacetate, phenylbutyrate, DCA or combination of phenylbutyrate and DCA. PDHC activity in brain, muscle, and liver mitochondrial fractions of mice treated with saline (n = 20), 250 mg/kg/day of phenylacetate (n = 5), 250 mg/kg/day of phenylbutyrate (n = 10), 25 mg/kg/day of DCA (n = 5), 250 mg/kg/day of DCA (n = 10), or with a combination of both drugs each at the dose of 250 mg/kg/day (n = 10). Averages ± SD are shown. a: p < 0.05 compared to saline-treated mice; b: p < 0.05 compared to 250 mg/kg/day of DCA; c: p < 0.05 compared to 250 mg/kg/day of phenylbutyrate. Averages ± SD are shown; * p < 0.05. *Abbreviations*: *PA*= phenylacetate; *DCA*= dichloroacetate; *PB*= phenylbutyrate

Phenylbutyrate is rapidly metabolized to phenylacetate in vivo (Mokhtarani et al 2012). In contrast to phenylbutyrate, phenylacetate does not affect PDHC activity in fibroblasts (Fig. 1) and does not increase enzyme activity in brain, muscle, and liver of wild-type mice (Fig. 2).

**Fig. 3:**
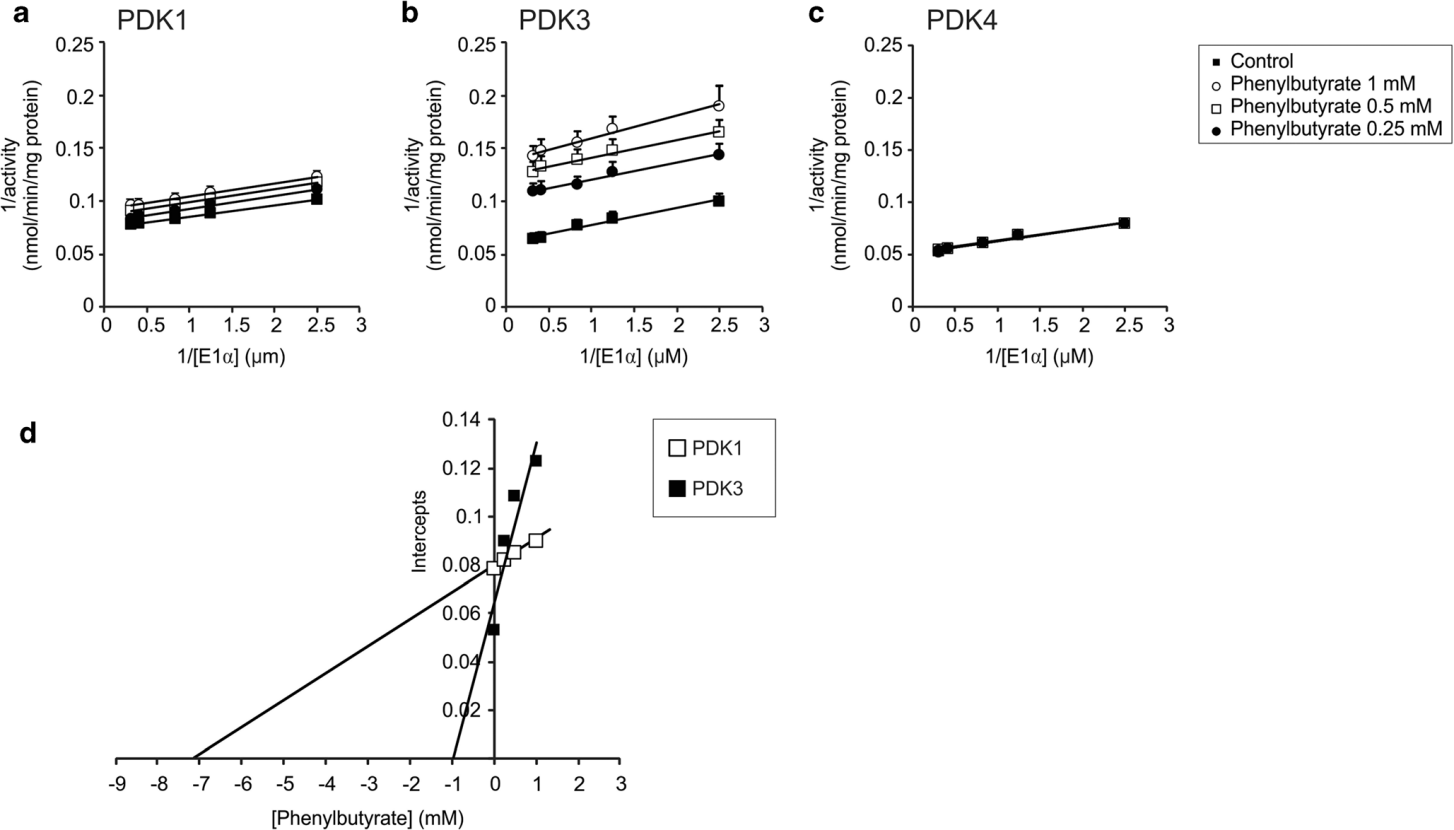
Differential inhibition of PDKs by phenylbutyrate. Lineweaver-Burk plots of PDK1 (**a**), PDK3 (**b**), PDK4 (**c**) in the absence (■) or in the presence of 0.25 mM (●), 0.5 mM (□), and 1 mM (○) of phenylbutyrate. Averages and standard errors of the mean are shown. (**d**) Secondary plots of the intercepts of lines from A and B against the relative inhibitor concentrations to compare the inhibitory effect of phenylbutyrate on different enzymes

We have previously reported that phenylbutyrate inhibition of PDK2 toward E1α substrate is reversible and competitive (Ferriero et al 2013). Here, we sought to investigate whether phenylbutyrate has inhibitory activity toward the other PDK isoenzymes (PDK1, PDK3, and PDK4) and the type of inhibition under the same conditions. These enzyme assays were performed on E1α substrate without E2, E1β, and other components of the complex. In Table 1, kinetic values of the four PDK isoenzymes obtained using recombinant PDK1, PDK2, PDK3, and PDK4 proteins are reported. Consistent with previous studies (Korotchkina and Patel 2001a, b), PDK2 is the isoenzyme with a much higher V_max_ compared to the other PDKs, whereas the four PDKs show little differences in their apparent K_m_, reflecting affinity for the E1α substrate. PDK1 and PDK3 were both inhibited by phenylbutyrate (Fig. 3a and b) whereas no inhibitory effect on PDK4 isoenzyme was observed (Fig. 3c). In primary Lineweaver-Burk plots, in the absence or in the presence of increasing concentrations of phenylbutyrate, a set of parallel and straight lines is obtained as a result of a concomitant but opposite effect of phenylbutyrate on K_m_ and V_max_ (Fig. 3a and b), thus showing uncompetitive inhibition on phosphorylation (Dixon and Webb 1979; Whiteley 2000). By plotting the values of intercepts on the *y* axis of the Lineweaver-Burk plot against inhibitor concentrations, the effect of phenylbutyrate on PDK1 and 3 can be compared (Fig. 3d). Compared to PDK1, the significantly lower K_i_ of phenylbutyrate for PDK3 (Table 1) indicates that phenylbutyrate is a stronger inhibitor of PDK3.

**Table 1:** Kinetic data of the four PDKs

	K_m_ (E1) mM	V_max_ nmol/min/mg protein	*k*_cat_ sec^−1^	K_i_ (phenylbutyrate) mM	Type of inhibition
PDK1	0.10 ± 0.015	12.90 ± 0.28	0.015 ± 0.001	7.1 ± 0.15	Uncompetitive
PDK2^§^	0.20 ± 0. 051	311.00 ± 42	0.363 ± 0.002	0.33 ± 0.08^§^	Competitive
PDK3	0.31 ± 0.028	16.86 ± 0.38	0.019 ± 0.0002	0.98 ± 0.19	Uncompetitive
PDK4	0.23 ± 0.021	19.79 ± 0.39	0.023 ± 0.063	N.D.	–

The assay was performed in a final pH pf 7.4 and phenylbutyrate was added at the concentration of 0.25 mM, 0.5 mM, or 1 mM. The enzyme concentrations of PDKs were 5.6-5.8 nM and PDK activity was measured as incorporation of [^32^P]-phosphate into E1α with 0.2 mM [*γ*-^32^P]ATP. K_i_ for DCA were previously found to be 1 mM for PDK1; 0.2 mM for PDK2; 8 mM for PDK3; and 0.5 mM for PDK4 (Bowker-Kinley et al 1998). K_m_ for ATP is not shown. Averages and standard errors of the mean are shown. ^§^From competitive inhibition (Ferriero et al 2013); *Abbreviations: N.D. = not determined*

### Docking studies

**Fig. 4:**
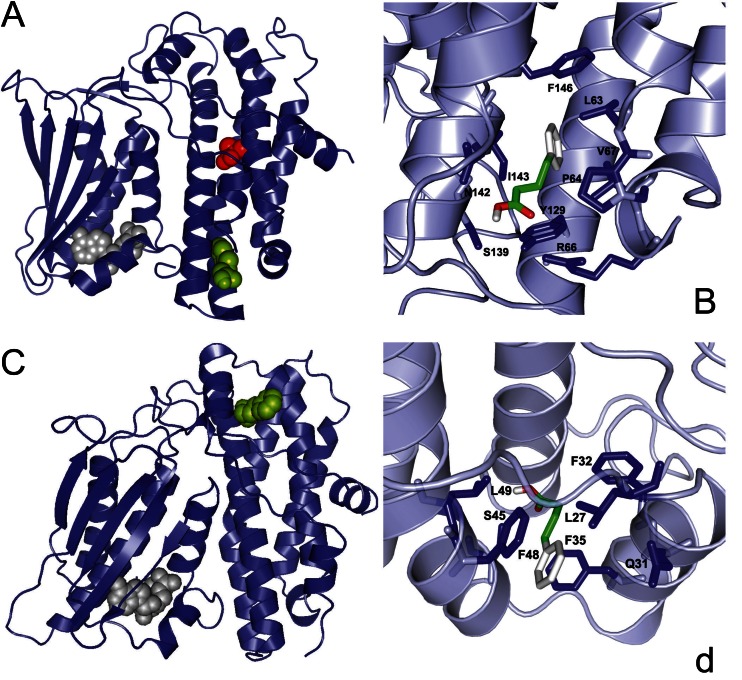
Putative binding sites of phenylbutyrate on PDK2 (**a**, **b**) and PDK3 (**c**, **d**). (**a**) Ribbon representation of human PDK2 structure (PDB 2BU8) and bound ligands represented in a space-filling model: ATP/Mg^2+^ in gray; phenylbutyrate in green; DCA in red. ATP and DCA are present in the X-ray structure while the phenylbutyrate position was suggested by docking analysis. (**b**) Specific interactions of phenylbutyrate (red, green, and white) with amino acid residues (stick representation) at the binding sites of PDK2. (**c**) Ribbon representation of human PDK3 structure (PDB 2Q8I) and bound ligands represented in a space-filling model: ATP/Mg^2+^ in gray; phenylbutyrate in green. ATP is present in the X-ray structure while phenylbutyrate binding site was suggested by the docking analysis. (**d**) Specific interactions of phenylbutyrate with amino acid residues (stick representation) of PDK3 at the binding sites. Van der Waals interaction spheres of the amino acid residues (stick representation) in contact with the inhibitor have been removed for clarity in **b** and **d**. Supplementary Fig. 1 shows Van der Waals interaction spheres of the amino acid residues in contact with the inhibitor

For PDK2, we previously identified by docking simulation two binding sites for phenylbutyrate: one near the ATP lid with a predicted binding affinity of −4.0 kcal/mol and the other at the base of the four helix cluster (Pfz3 binding site) (Knoechel et al 2006) with a predicted binding affinity of −5.6 kcal/mol (Ferriero et al 2013). The PDK2 structure used for this simulation was an ATP-free structure (PDB ID 2BU7) and because of the high affinity of PDK2 for ATP, we here performed a docking simulation of phenylbutyrate toward the ADP-bound structure of PDK2 (PDB ID 2BU8) to simulate conditions occurring both in vitro and in vivo. When the ATP binding site is occupied, only the Pfz3 binding site was identified with an even higher binding affinity (−6.9 kcal/mol) in the PDK2 structure compared to the PDK2 ATP-free form (−5.6 kcal/mol) (Ferriero et al 2013). This result suggests that this region in PDK2 is likely to be the most relevant binding site involved in phenylbutyrate inhibition. This binding pocket for phenylbutyrate is located at one of the highly lipophilic four-helix bundle of PDK2 (Fig. 4a). Val67 located in the middle of this pocket is likely to play an important role in stabilizing binding to phenylbutyrate (Fig. 4b and Supplementary Fig. 1a). Interestingly, this residue is unique to human PDK2 whereas at that amino acid position the other three isoforms have a leucine that has a larger side chain which could restrict the access of phenylbutyrate to the binding pocket. Docking of phenylbutyrate on a PDK2 model in which Val67 was replaced in silico with leucine indeed resulted in lack of drug binding at this site.

Because of the stronger inhibitory effect and its importance as an anti-cancer target (Lu et al 2008; Kluza et al 2012), we searched for phenylbutyrate binding site on PDK3. Through a docking simulation, we identified a putative binding site in the N-terminal region of PDK3 (PDB 2Q8I) with a binding affinity of −7.2 kcal/mol (Fig. 4c). This binding site consists of a narrow hydrophobic channel formed by Leu27, Gln31, Phe32, Phe35, Ser45, and Phe48 residues (Fig. 4d and Supplementary Fig. 1b).

### Combined treatment with phenylbutyrate and DCA

**Fig. 5:**
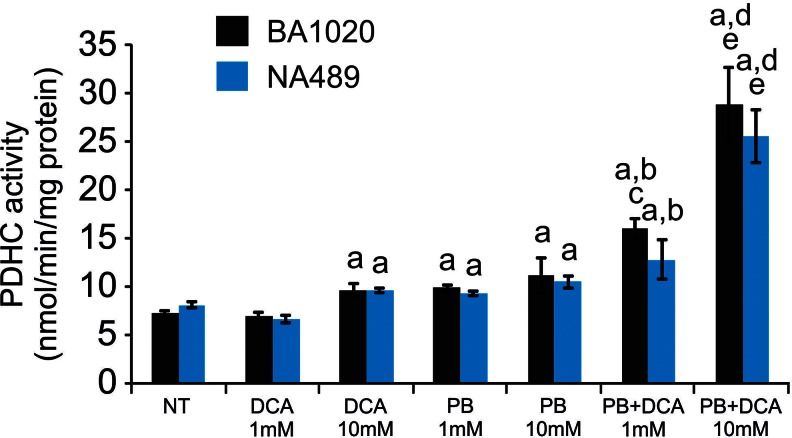
PDHC activity in wild-type fibroblasts incubated with phenylbutyrate, DCA, or a combination of phenylbutyrate and DCA. PDHC activity expressed as nmol/min/mg protein of mitochondrial fractions of two wild-type cell lines (BA1020 and NA489) incubated with 1 mM or 10 mM DCA, 1 mM or 10 mM of phenylbutyrate, with the simultaneous presence of 1 mM DCA and 1 mM phenylbutyrate, or with the simultaneous presence of 10 mM DCA and 10 mM phenylbutyrate. Averages ± SD are shown. a: p < 0.05 compared to N.T.; b: p < 0.05 compared to DCA 1 mM; c: p < 0.05 compared to phenylbutyrate 1 mM; d: p < 0.05 compared to DCA 10 mM; e: p < 0.05 compared to phenylbutyrate 10 mM. *Abbreviations*: *NT*= not treated (no drug); *DCA*= dichloroacetate; *PB*= phenylbutyrate

**Fig. 6:**
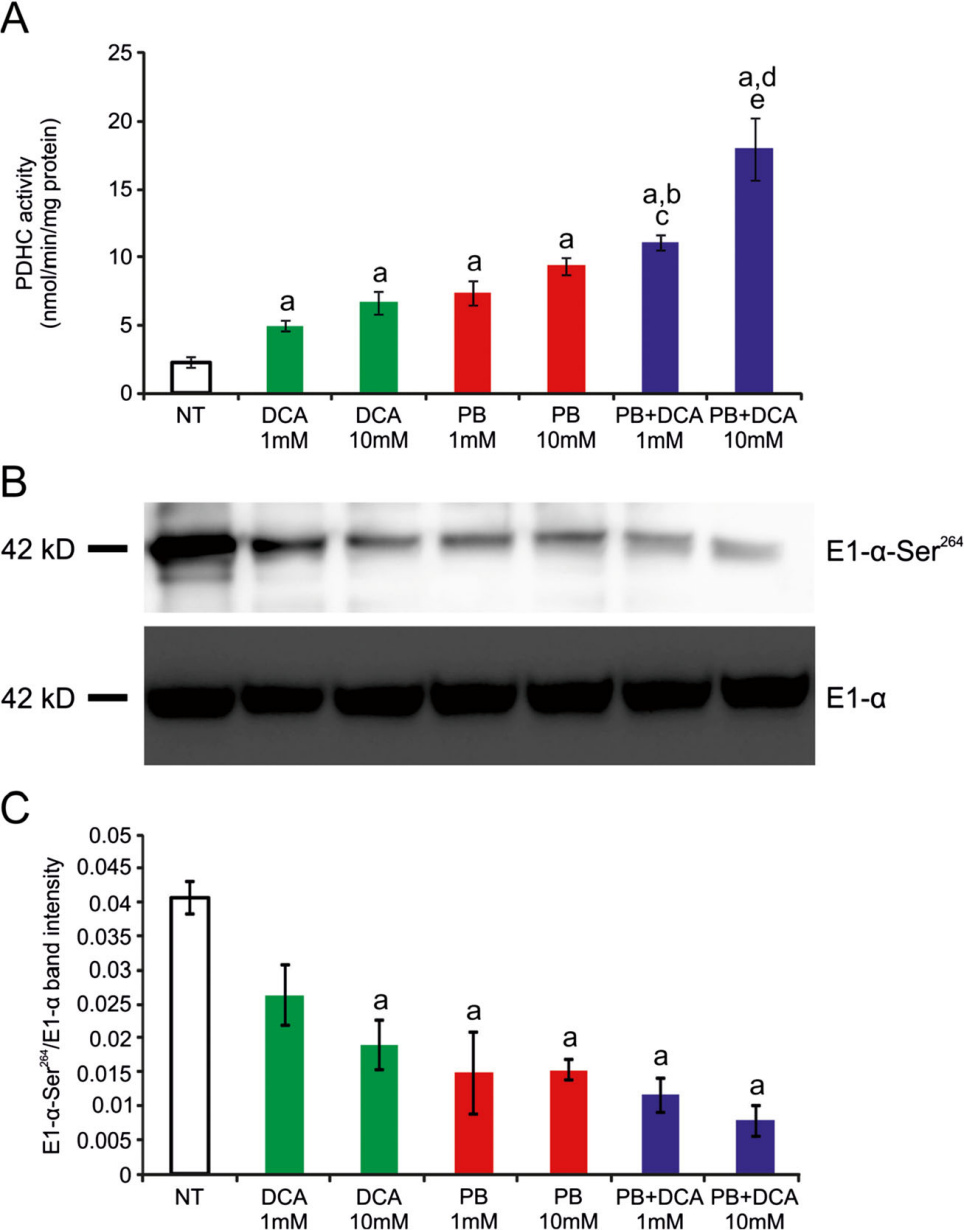
PDHC activity and phosphorylation of PDHC deficient cells incubated with phenylbutyrate, DCA, or a combination of phenylbutyrate and DCA. (**a**) PDHC activity expressed as nmol/min/mg protein in a PDHC deficient patient harboring the mutation p.N135S in the *PDHA1* gene incubated with 1 mM or 10 mM DCA, 1 mM or 10 mM of phenylbutyrate, with the simultaneous presence of 1 mM DCA and 1 mM phenylbutyrate, or with the simultaneous presence of 10 mM DCA and 10 mM phenylbutyrate. Averages ± SD are shown. a: p < 0.05 compared to N.T.; b: p < 0.05 compared to DCA 1 mM; c: p < 0.05 compared to phenylbutyrate 1 mM; d: p < 0.05 compared to DCA 10 mM; e: p < 0.05 compared to phenylbutyrate 10 mM. (**b**) Western blotting for phosphorylated E1α and for total E1α protein in cells incubated with the conditions reported in panel **a**. The images are representative of two independent experiments. (**c**) Relative band intensities of phosphorylated E1α normalized for total E1α from two independent experiments. Averages ± SD are shown. *Abbreviations*: *DCA*= dichloroacetate; *PB*= phenylbutyrate; *NT*= not treated (no drug)

Because phenylbutyrate is predicted to bind to the ubiquitously expressed PDK2 on a different site compared to DCA (Fig. 4a and b), a known PDK inhibitor (Stacpoole 1989), we hypothesized that co-administration of both drugs results in increased PDHC enzyme activity. To test this hypothesis, we first incubated wild-type human fibroblasts with a combination of DCA and phenylbutyrate or with DCA and phenylbutyrate alone as controls. A greater increase in PDHC activity was detected in two independent wild-type fibroblast cell lines incubated simultaneously with 1 mM or 10 mM of phenylbutyrate and DCA compared to the sum of the effects of each drug alone (Fig. 5, p < 0.05). An increase of PDHC activity was also observed in fibroblasts from a patient with PDHC deficiency harboring the p.N135S mutation in *PDHA1* gene (Ferriero et al 2013) (Fig. 6a, p < 0.05). Consistent with previous study (Ferriero et al 2013), this fibroblast cell line responded to phenylbutyrate with a significant increase of enzyme activity that was also increased by DCA and was further enhanced by a combination of the two drugs (Fig. 6a). Phenylbutyrate and DCA also resulted in reduction of phosphorylated E1α and such reduction was greater in cells incubated with a combination of the two drugs (Fig. 6b and c). Wild-type mice administered orally by gavage with a combination of 250 mg/kg/day of DCA and 250 mg/kg/day of phenylbutyrate showed higher PDHC enzyme activity in brain, muscle, and liver mitochondrial fractions compared to mice administered with each drug alone (Fig. 2, p < 0.05). DCA administered in mice at the dose of 25 mg/kg/day, that is the dose administered in clinical trials in patients with inherited PDHC deficiency (Stacpoole et al 2006), resulted in no significant increase of PDHC activity in the analyzed tissues (Fig. 2).

## Discussion

PDK isoforms are up-regulated in cancer, diabetes, obesity, and heart failure and are potential therapeutic targets for these diseases (Roche and Hiromasa 2007). Moreover, PDK inhibition results in increased PDHC activity that has potential for treatment of patients with inherited PDHC deficiency for which no proven effective treatments are currently available (Stacpoole et al 1997). In this study, we show that phenylbutyrate and not its bioproduct phenylacetate is effective at increasing PDHC activity. These results are consistent with a previous study showing that phenylbutyrate and not phenylacetate binds BDK with high affinity and strongly interferes with its enzymatic activity (Tso et al 2013). In vivo phenylbutyrate is rapidly converted into phenylacetate. In humans, following oral administration, phenylbutyrate concentration reaches a peak in the serum at 1.5 hours and becomes undetectable within about 6 hours (Yu et al 2001). Therefore, as for urea cycle disorders, multiple daily administrations will likely be required to achieve sustained increase of residual PDHC activity in vivo.

We investigated the inhibitory activity of phenylbutyrate toward PDKs that negatively regulate PDHC by reversible phosphorylation. Enzyme assay was performed using purified E1α and thus, we cannot rule out that results of kinetic studies with PDKs may be different if the whole E1 heterotetramer is used as a substrate. Nevertheless, PDK isoforms 1-to-3 were found to be inhibited by phenylbutyrate. PDK2 and PDK3 were more strongly inhibited compared to PDK1 (Table 1) and PDK2, that has the highest V_max_ and lowest K_i_ (Table 1), is likely to be the main target for phenylbutyrate in vivo. In contrast, PDK4 that was previously proposed as a lipid-responsive PDK isoform (Sugden and Holness 2003) was not inhibited. Therefore, in addition to BDK, we identified PDK1-to-3 as additional molecular targets of phenylbutyrate (Brunetti-Pierri et al 2011; Tso et al 2013).

Structural differences among the PDKs might explain the different inhibitory effect of phenylbutyrate toward PDK2 and the other isoenzymes. The binding site in PDK2 is predicted to be located far from the E1α substrate binding site and the competitive inhibition might be explained by conformational changes induced by phenylbutyrate on PDK2 that reduce only its affinity toward the E1α without affecting the *k*_cat_. The binding affinity of PDK3 for E1α is strongly affected by binding to L2 domain of E2 and in the absence of such binding PDK3 is less active (Baker et al 2000; Korotchkina and Patel 2001b). Uncompetitive inhibition is observed when the inhibitor binds the enzyme-substrate complex in a site that is different from the active site but induces a conformational change that affects affinity and activity of the enzyme for the substrate (Dixon and Webb 1979). We hypothesize that binding of phenylbutyrate to its putative site identified by docking on PDK3 interferes with E1α binding through conformational changes that negatively affect the catalytic activity. This hypothesis can support the uncompetitive inhibition that is expected to be stronger in the presence of E2, which was absent in our assay. The pocket involved in phenylbutyrate binding is only present in PDK3 and is part of a region involved in binding to the L2 domain of E2 subunit (Kato et al 2005). Additional studies, such as X-ray crystallography of PDK-phenylbutyrate complex, are required to confirm binding of phenylbutyrate to the site predicted by the docking studies.

To explain the lack of inhibition of phenylbutyrate toward PDK4, we compared the available structures of PDK2 and PDK4 in their ADP-bound form. The PDK2-ADP bound form (PDB 2BU8) results in a closed conformation with disordered C-term tails. In this form, the ATP lid is structured and the protein has a high affinity for ATP. In contrast, the PDK4-ADP bound form (PDB 2ZKJ) has an intermediate open conformation in which the ATP lid is unstructured and cross tails are partially ordered (Wynn et al 2008). This form has lower affinity for ATP and the ordered cross tails may impair entrance of phenylbutyrate in the structure. Different or even opposite effects on PDKs have also been found with Nov3r ligand (Knoechel et al 2006). Although binding to L2 domains of E2 subunits on both PDK2 and PDK4, Nov3r inhibits PDK2 but also stimulates PDK4 activity. The non-conservative change in Leu32 of PDK4, that affects affinity of the ligand for L2 domain, might explain the different effects occurring upon binding of Nov3r (Knoechel et al 2006).

PDHC is a key enzyme in metabolism and plays an important role in cancer. Through PDHC inhibition, PDK1 and PDK3 are involved in hypoxia-induced metabolic switch from aerobic respiration to aerobic glycolysis (i.e., the Warburg effect) thus promoting cell survival (Kim et al 2006; Lu et al 2008; Kluza et al 2012; Ferriero and Brunetti-Pierri 2013). Inhibition of PDK1 or PDK3 impairs cell growth and increases oxygen consumption in human cancer cell lines (Kim et al 2006; Lu et al 2008; McFate et al 2008). Moreover, PDK1 depletion is effective in eradicating cancer by activation of oncogene-induced senescence (Kaplon et al 2013). Therefore, PDK1 and PDK3 inhibition by phenylbutyrate might be therapeutically effective in cancer. Phenylbutyrate has indeed shown efficacy in different cancers (Iannitti and Palmieri 2011). Similarly, DCA has also shown efficacy in several different types of human cancers (Michelakis et al 2008, 2010).

Recently, a mutation conferring PDK3 hyperactivity has been reported as a cause of Charcot-Marie-Tooth disease (Kennerson et al 2013) and by inhibition of PDK3 activity, phenylbutyrate may prevent PDHC hyper-phosphorylation that has been recognized as the underlying cause of peripheral neuropathy. In addition, phenylbutyrate might be effective for therapy of patients with inherited PDHC deficiency, an inborn error of metabolism that severely affects the central nervous system (Brown et al 1994; DeBrosse et al 2012).

In this study, we have shown that a combination of phenylbutyrate and DCA results in greater increase of PDHC activity compared to each drug alone. DCA has been proposed as a treatment for patients with inborn errors of mitochondrial metabolism (Coude et al 1978) and its efficacy has been investigated in clinical trials. A randomized controlled trial of DCA at the dose of 25 mg/kg/day in children with PDHC deficiency or defects in respiratory chain enzymes showed good tolerance and sustained reductions of venous blood and cerebrospinal fluid lactate concentrations (Stacpoole et al 2006). However, some concerns were raised because of worsening of lower extremity nerve conduction in some patients (Stacpoole et al 2008), particularly in adults (Kaufmann et al 2006).

In mice, DCA and phenylbutyrate both increased PDHC activity in brain, muscle, and liver at the dose of 250 mg/kg/day whereas no increase of enzyme activity was detected with 25 mg/kg/day of DCA that corresponds to the dose administered to patients with cancer (Dunbar et al 2014) and PDHC deficiency (Stacpoole et al 2006). It is unknown whether higher dose of DCA are needed to obtain clinically relevant outcomes and whether such higher doses are tolerated in humans. Nevertheless, DCA is metabolized in humans at a lower rate compared to mice and high doses might not be required (Stacpoole et al 1998). Phenylbutyrate instead was effective in mice at increasing PDHC enzyme activity at the dose of 250 mg/kg/day that is safely administered to patients with urea cycle disorders (Haberle et al 2012). The results of our study show that combination of phenylbutyrate and DCA has the potential of resulting in greater efficacy compared to the single drug treatments. However, careful evaluation of toxicity deriving from this drug combination would be required prior to testing in patients.

In conclusion, the greater increase of PDHC activity both in cells and in vivo suggests that combined therapy with drugs binding to different PDK sites, such as phenylbutyrate and DCA, has potential to enhance therapeutic efficacy by greater enhancement of enzyme activity. Such combined therapy could be applied for therapy of patients with cancer, PDHC deficiency, and several other disorders that can benefit from PDK inhibition.

## Electronic supplementary material

Below is the link to the electronic supplementary material.

ESM 1 (PDF 112 kb)
