# Supplementary material for: Differential inhibition of PDKs by phenylbutyrate and enhancement of pyruvate dehydrogenase complex activity by combination with dichloroacetate
Source: J Inherit Metab Dis. 2015 Jan 20;38(5):895–904. doi: 10.1007/s10545-014-9808-2 (PMC4551558; doi:10.1007/s10545-014-9808-2)
Supplement: Supplementary file 1 — (PDF 112 kb) [file 10545_2014_9808_MOESM1_ESM.pdf]

Differential inhibition of PDKs by phenylbutyrate and enhancement of pyruvate dehydrogenase complex activity by combination with dichloroacetate.

Rosa Ferriero, Clara Iannuzzi, Giuseppe Manco, and Nicola Brunetti-Pierri.

Supplementary Figure 1.

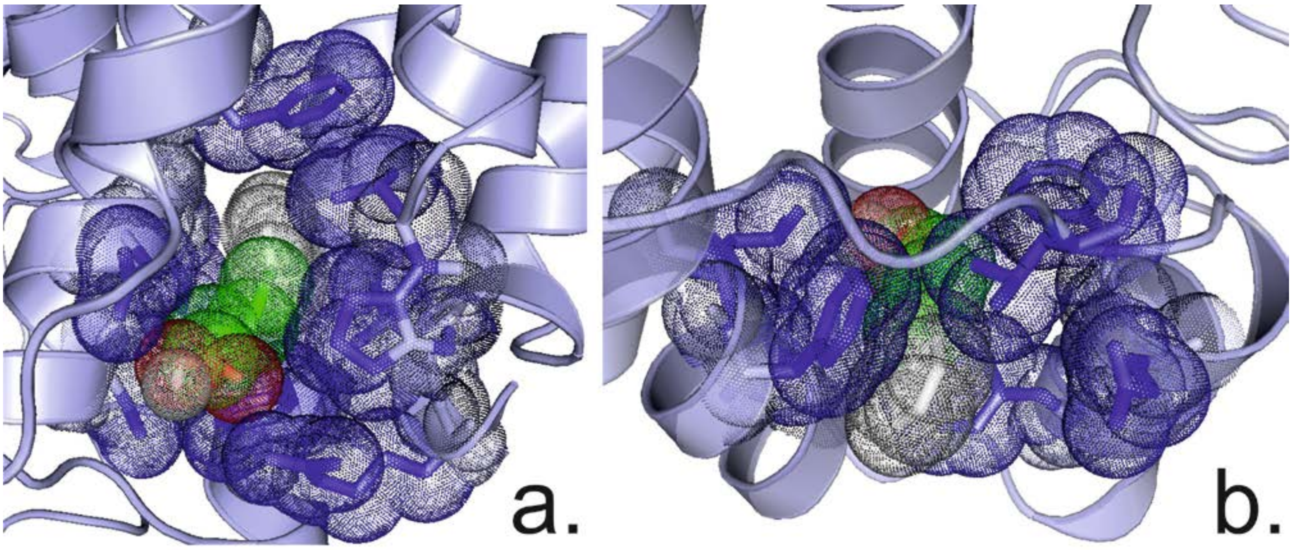

Supplementary Figure 1. Putative binding sites of phenylbutyrate on PDK2 (**a**) and PDK3 (**b**). Interactions of phenylbutyrate with amino acid residues at the binding sites include Van der Waals interaction spheres of amino acid residues in contact with the inhibitor.
